# Supplementary material for: VGsim: Scalable viral genealogy simulator for global pandemic
Source: PLoS Comput Biol. 2022 Aug 24;18(8):e1010409. doi: 10.1371/journal.pcbi.1010409 (PMC9447924; doi:10.1371/journal.pcbi.1010409)
Supplement: S1 File — Supporting information file with the introduction to epidemiological compartmental models, details of the migration model, presentation of the simulation procedure and algorithms, example of the software Python API, details on the implemented sampling strategy, validation of the software implementation and detailed discussion of other epidemiological simulators. (PDF) [file pcbi.1010409.s001.pdf]

VGsim: scalable viral genealogy simulator for global pandemic. Supplementary information

Vladimir Shchur*[1], Vadim Spirin[1], Dmitry Sirotkin[1], Evgeni Burovski[2], Nicola De Maio[3], and Russell Corbett-Detig[4]

[1]International laboratory of statistical and computational genomics, HSE University, Moscow, 101000, Russia

[2]HSE University, Moscow, 101000, Russia

[3]European Molecular Biology Laboratory, European Bioinformatics Institute, Wellcome Genome Campus, Hinxton, Cambridgeshire, CB10 1SD, UK

[4]Department of Biomolecular Engineering and Genomics Institute, UC Santa Cruz, California 95064, USA

June 14, 2022

1 Introduction to compartmental models

To get some basic intuition behind the compartmental models used in the forward pass, consider the simple case of a single population, a single viral haplotype, and an SIS compartmental model. In this model every individual can

*Corresponding author: vshchur@hse.ru

be either susceptible (S) or infectious (I). If a susceptible individual meets an infectious individual, there is a chance x that the infectious individual infects the susceptible one. All individuals have the same contact rate y (the number of other individuals they meet in a time unit). Assume there are $|S(t)|$ and $|I(t)|$ susceptible and infectious individuals at time t, and the constant total population size is N. For a single susceptible individual the chance that the next random encounter with another person would be with an infectious individual is $|I(t)|/(N-1)$ (though N is usually large, so $N-1 \approx N$). So the rate at which a single susceptible individual becomes infected is $\lambda' = \lambda \frac{|I(t)|}{N} xy$ (contact rate times the probability that the second individual is infectious times the probability of transmission). The total rate of a new infection in the population will be $\tilde{\lambda} = |S(t)|\lambda'$. Similarly, infectious individuals recover with some rate ρ, and the total recovery rate in the population is $\tilde{\rho} = \rho|I(t)|$. From the theory of Poisson processes, it follows that the total rate of events in the population is simply the sum $\tilde{\lambda} + \tilde{\rho}$, and the waiting time of the first event is distributed exponentially with rate (parameter) $\tilde{\lambda} + \tilde{\rho}$, and with probability $\tilde{\lambda}/(\tilde{\lambda} + \tilde{\rho})$ the next event is an infection. This gives a straightforward method to simulate continuous-time epidemiological dynamics under this model. This approach is easily generalised to an arbitrary number of compartments. The SIS model is the basis for all the compartmental models we implemented, and in particular we expand on the basic model (as described below in details) by allowing different types of S and I compartments in the same simulation.

2 Migration

In our model, migrations are interpreted as visits of individuals from one population into another. The expected number of individuals present at any time in

population s is

$$N_f^s = \sum_r \mu_{rs} N^r,$$

where N^r is the number of individuals native to population r, μ_{rs} is the probability to find an individual native of population r in population s (notice that $\mu_{ss} = 1 - \sum_{r \neq s} \mu_{sr}$). This model is accurate in the case of large population sizes. For small populations, agent-based simulators are more appropriate (see SI 9).

The rate of transmission of haplotype h to an individual with immunity i within population s is

$$\sum_q \lambda_h \sigma_{ih} \delta^q \left(\mu_{sq} |I_h^s|\right) \frac{\mu_{sq} |S_i^s|}{N_f^q} = \lambda_h \sigma_{ih} |I_h^s| |S_i^s| \sum_r \frac{\delta^q \mu_{sq}^2}{N_f^q}, \tag{S.1}$$

where, we remind, λ_h is the base transmission rate of the haplotype h, σ_{ih} is the susceptibility of immune group i to haplotype h, δ^s is the contact density within population s, and μ_{ss} is the probability that an individual native to population s is in their home population at the time of the event. Each term of the sum corresponds to a population q where two individuals from the population s interact.

3 Efficient algorithm for migrations

Instead of updating every particular propensity for infectious and susceptible compartments from different populations, we rely on an upper-bound for propensities corresponding to migration events. If a potential migration is sampled, the algorithm then calculates the actual propensity and accepts or rejects the migration event with probability equal to the ratio of the actual propensity and its upper bound.

Here we derive the upper bounds on migration rates. For this purpose, we

set $\Sigma_h = \max_i \sigma_{ih}$, $\Lambda = \max_h \lambda_h \Sigma_h$. Then the following holds

$$\sum_{i,h} M(r,i,s,h) \leq \Lambda \sum_{i,h,q} |S_i^r||I_h^s| \frac{\mu_{sq}\mu_{rq}\delta^q}{N_f^q}$$
$$= \Lambda S^r I^s M_{sr},$$

where $M(r,i,s,h)$ is defined by equation 1 (main text), $S^r = \sum_i |S_i^r|$ and $I^s = \sum_h |I_h^s|$. Denote $M_{sr} = \sum\limits_q \frac{\mu_{sq}\mu_{rq}\delta^q}{N_f^q}$. So, the total migration (or introduction) rate from source population s into target population r is given the upper bound of $\Lambda M_{sr} S^r I^s$.

We finally get the upper bound for the total migration rate:

$$\sum_{s,r:s\neq r} \Lambda M_{sr} S^r I^s = \Lambda \sum_r S^r \sum_{s:s\neq r} M_{sr} I^s \leq \Lambda \sum_r S_r (\max_s M_{sr})(I_g - I_r),$$

where S_g and I_g are the total number (over all populations) of susceptible and infectious individuals in the simulation.

If the algorithm samples a potential migration, it samples s, r, h and i with the probabilities $\frac{|S_i^r||I_h^s|}{S_g I_g}$, and accepts the migration with probability $\frac{\lambda_h \sigma_{ih}}{\Lambda \max_{s,r} M_{sr}}$. Otherwise, the migration is discarded and the algorithm proceeds to the next iteration. The logical basis of this approach follows from the additivity of Poisson processes (similarly to the reasoning behind the standard Gillespie algorithm [1]).

4 Forward run

The forward run consists of sampling events which determine the development of the epidemiological process at the population level. In order to efficiently sample events using the Gillespie algorithm, one needs to iteratively 1) sample

an event given the reaction propensities, 2) update the reaction propensities.

Event sampling is organised similarly to the logarithmic direct method [1] which groups all the reactions in subsets which can be represented as a tree. We split the reactions by the following logic represented in Figure 1

- First, the algorithm chooses between a migration event or a within-population event.
- For migration events the following parameters are chosen subsequently: target population (where the new introduction occurs), source population of the infection, introduced haplotype, immunity type of the individual contracting the infection, rejection or acceptance of the migration event (see below).
- All other events are considered to be within-population; for these, first the population of the event is sampled.
- Then, choose if the event is a susceptibility transition.
- For susceptibility transitions, choose the source and target susceptibility groups.
- Otherwise, choose the infectious compartment (haplotype) involved in the event.
- Finally, choose the type of event: recovery, sampling, mutation or transmission.
- For mutations: the genomic site and the nucleotide substitution are chosen subsequently.
- For transmissions: the immunity type of the individual being infected is chosen.

As we explained in SI 3, instead of keeping propensities for each infectious-susceptible interaction between populations, we keep track of their upper bound and apply a rejection-based algorithm [2] to the sampling of migration events.

We cache the total propensities of each population at each step of the algorithm. We also keep track of the propensity dependencies, similarly to the optimised direct algorithm [3], to avoid updating propensities which are not affected by the reactions.

5 Backward run

The backward run is carried out backward in time, starting from the last sampling event simulated during the forward run. $|I_h^s|$ is again the total number of individuals carrying haplotype h in population s at a given time. Denote by $\mathbb{L}_h^s$ the set of sample ancestral lineages with haplotype h in population s at the considered time, and define $L_h^s = |\mathbb{L}_h^s|$ its size.

A new infection forward in time corresponds to a coalescence between two lineages backward in time. Becoming non-infectious the forward in time corresponds to a recovered individual becomes infectious backward in time. Mutations simply revert from the derived allele back to ancestral allele when considered backward in time. Migration-associated transmission correspond backward in time to coalescent events between lineages in different populations.

A more detailed list of possible events for the backward run and their rates is the following:

- New infection with haplotype h in population s. Two sample ancestral lineages coalesce with probability

$$\frac{\binom{L_h^s}{2}}{\binom{|I_h^s|}{2}},$$

which is the ratio of the number of pairs of lineages over the number of pairs of infected individuals. If indeed a coalescent event is sampled, we randomly choose and remove a pair of lineages l_1, l_2 from $\mathbb{L}_h^s$, add an ancestral node l_a into the sample genealogy, and set the parent of l_1 and l_2 to l_a. In any case $|I_h^s| \leftarrow |I_h^s| - 1$.

- Recovery of an individual infected with haplotype h in population s. Becoming non-infectious corresponds to a recovered individual becoming infectious backward in time. Hence, $|I_h^s| \leftarrow |I_h^s| + 1$.

- Sampling an individual infected with haplotype h in population s. As with recovery, $|I_h^s| \leftarrow |I_h^s| + 1$. Also, a new leaf node is added to the genealogy, and the corresponding lineage is added into the set $\mathbb{L}_h^s$.

- Mutation transforming haplotype h into derived haplotype m in population s. This mutation happens to a sample ancestral lineage with probability $L_m^s/|I_m^s|$. In this case we randomly choose a lineage from L_m^s and move it into L_h^s. In any case update

$$|I_m^s| \leftarrow |I_m^s| - 1$$
$$|I_h^s| \leftarrow |I_h^s| + 1.$$

- Transition between susceptible compartments (immunity change). This does not have any immediate effect on the genealogy.

- Migration of haplotype h from source population s into target population r. First, with probability $L_h^r/|I_h^r|$ a sample ancestral lineage is affected by the migration. In this case we randomly choose a lineage from $\mathbb{L}_h^r$ and move it into $\mathbb{L}_h^s$. With probability $(L_h^s - 1)/|I_h^s|$ this lineage coalesces with a sample ancestral lineage in the source population. In this case

we randomly draw a lineage from $\mathbb{L}_h^s$ (except for the one which was just moved there), and update the genealogy similarly to the case of a new infection. In any case update

$$|I_h^r| \leftarrow |I_h^r| - 1$$

6 VGsim's Python API

VGsim's API allows initializing such a model with thousands of reactions in a few lines of Python code. With $U = 2$ mutable sites, $K = 100$ populations and $T = 3$ susceptibility types, there are more than $5 \cdot 10^5$ reactions.

```
import VGsim
number_of_sites = 2
populations_number = 100
number_of_susceptible_groups = 3
simulator = VGsim.Simulator(number_of_sites, populations_number,
    number_of_susceptible_groups, seed=1234)
```

Listing 1: VGsim initialisation

Now we set the transmission ($\lambda = 2.5$), recovery ($\rho = 0.99$), sampling ($\zeta = 0.01$) and mutation ($m = 0.00002$) rates, and the susceptibility type ($i = 1$, enumeration starts from 0) after recovery for all the haplotypes. These rates correspond to SARS-CoV-2 wild-type parameters if time measured in a unit of one week.

```
simulator.set_transmission_rate(2.5)
simulator.set_recovery_rate(0.99)
simulator.set_sampling_rate(0.01)
simulator.set_mutation_rate(0.00002)
simulator.set_susceptibility_type(1)
```

Listing 2: Setting baseline rates.

Then the parameters of this model can be fine-tuned (from now on all the parameters might be non-realistic and used only to demonstrate different features of the API). In the next few lines we set the following features: a transmission rate $\lambda = 4.0$ of all haplotypes carrying allele G, migration probabilities $\mu_{sr} = 10/365$ assuming every individual spends on average 10 days outside their population of origin, vaccination efforts corresponding to susceptibility transitions from types 0 and 1 into type 2.

```
simulator.set_transmission_rate(4.0, haplotype='G*')
simulator.set_migration_probability(total_probability=10/365)
simulator.set_immunity_transition(0.35, target=2)
```

Listing 3: Adjusting the simulated model.

Finally we tune the susceptibility. There will be partial immunity $\sigma_1 = 0.2$ after recovery to all haplotypes except for 'GT'. Previous infection increases the susceptibility to haplotype 'GT' by 10%: $\sigma_{i,GT} = 1.1$. Vaccine gives complete resistance to all haplotypes. Finally, run the simulation for 10 time units and no more than 10^7 events.

```
simulator.set_susceptibility(0.2, susceptibility_type=1)
simulator.set_susceptibility(1.1, susceptibility_type=1, haplotype=
    'GT')
simulator.set_susceptibility(0.0, susceptibility_type=2)
simulator.simulate(10000000, time=10)
```

Listing 4: Setting immunity model.

More details can be found in the documentation and tutorials at `https://vg-sim.readthedocs.io/`)

7 Sampling

Sampled hosts are considered to be recovered and they are moved to a susceptible group (corresponding to the immunity triggered by the pathogen strain).

Sampling rates can be adjusted in each population to reflect differences in the sampling/sequencing efforts. Sampling rates can be also changed over time through the Python API between consequent calls of the `simulate()` method similarly to changing other parameters. It can be used, for example, to define the scenario where sampling begins with a delay after the pathogen first appears in the population.

We also note that it is straightforward for users to prune samples from the genealogy at any point in the simulation using matUtils [4] if this is thought to reflect a more realistic sampling scheme.

8 Correctness of the software implementation

We used `MASTER` [5] to verify the consistency of epidemiological trajectories and genealogies simulated with `VGsim`.

We implemented a model that uses most of the features of `VGsim`:

- two populations (demes) with $100,000$ individuals in each of them, migration probability of 0.1 (symmetric),
- one mutable genetic site with allele G leading to increased transmissibility,
- three immunity groups
  - fully susceptible,
  - immunity triggered by alleles A, T and C: full resistance against these alleles, partial resistance against allele G,
  - immunity triggered by allele G: full resistance against all the alleles.

- loss of triggered immunity with time.

The corresponding MASTER model comprises a total of 56 reaction equations: in each of two population there are $4+1$ new infection reactions, $4+1$ migration reactions, 4 recovery reactions, $3*4$ mutation reactions, 2 triggered immunity loss reactions.

We generated 100 replicates with both VGsim and MASTER using the exact Guillespie algorithm. The epidemiological trajectories are shown in Figures 1 and 2. The high variance of these trajectories is explained by the fact that mutations are rare events in this model.

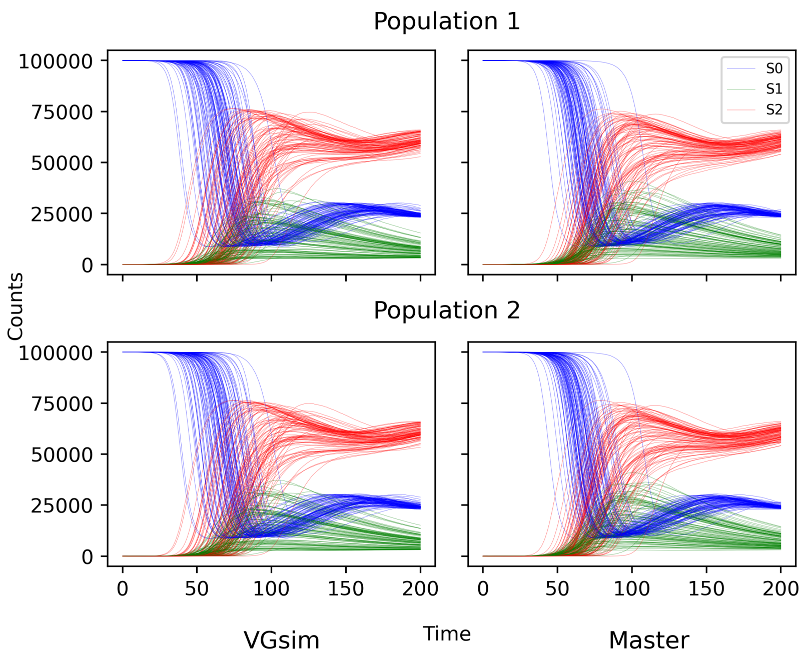

Figure 1: Stochastic trajectories of the number of susceptible individuals simulated with VGsim (left column) and MASTER (right column). $S0$ is the initial susceptible type (no resistance to any of four haplotypes). $S1$ is the immunity triggered by an infection with pathogen haplotypes 'A', 'T' and 'C'. $S2$ is the immunity triggered by haplotype 'G'.

Further, in order to validate the correctness of genealogies, we ran 100 repli-

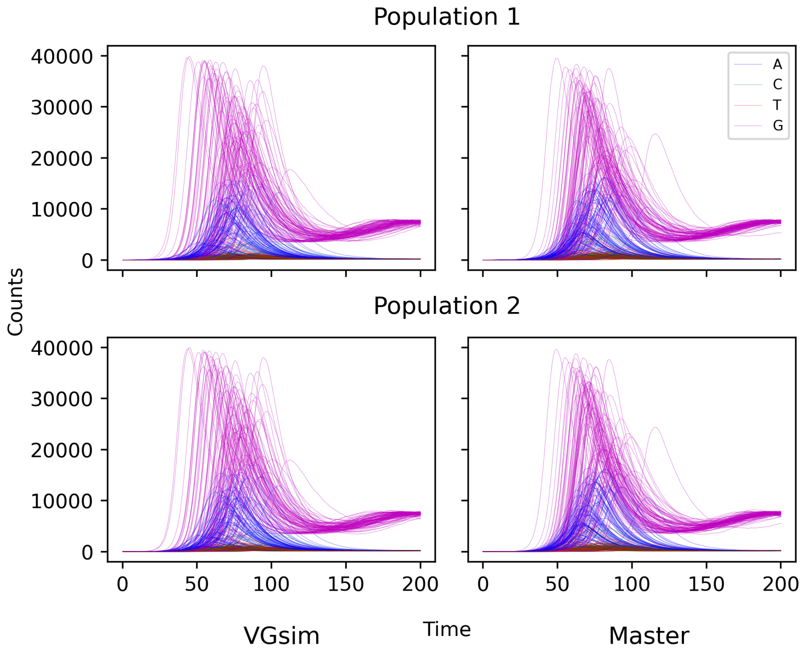

Figure 2: Stochastic trajectories of the number of infectious individuals simulated with `VGsim` (left column) and `MASTER` (right column) carrying different pathogen haplotypes.

cates of this simulation to generate viral genealogies and estimated the distribution of coalescent times for each genealogy. We also calculated the average distribution of all the replicates. The results are shown on Figure 3.

Here we give more details on the model's parameters values.

We consider a two-deme model with migration probability 0.1 and a pathogen with 1 mutable site. The average time for a person to recover is set to be equal to 10 days. Strains, corresponding to the haplotypes "A", "T" and "C" have the same rate of infection, which correspond to 2.5 infected persons from one infectious in the beginning of the pandemics throughout the disease. The strain corresponding to haplotype "G" is more virulent and results in 4 infected individuals on average. The mutation rate is assumed to be equal 0.003 with

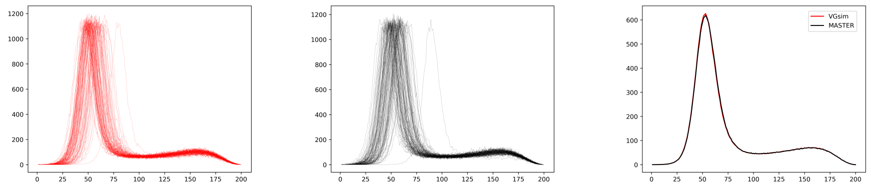

Figure 3: The distributions of coalescent times from genealogies generated by `VGsim` on the left panel and `MASTER` on the middle panel. Averages of these distributions for `VGsim` and `MASTER` are shown on the right panel.

uniform distribution between each strain. Individuals who recovered from the illness with haplotypes "A", "T" and "C" are immune to all haplotypes but "G" (although, they are 5 times more resistant to it). Individuals who recovered from the illness with haplotype "G" are fully immune. Immunity is gradually lost and individual becomes fully susceptible to the pathogen after 100 days on average.

The code, which sets this model in `VGsim` is available on `https://github.com/Genomics-HSE/VGsim/tree/main/example`

9 `VGsim` and other epidemiological simulators

Several epidemiological simulators were developed in recent years [6–14] to address many specific scenarios, each of which required certain trade-offs of general applicability vs realism and computational efficiency.

For example, agent-based simulators are able to tune models with extreme precision based on the exact properties of each agent. They also can model additional complex agent interactions — for example `nosoi` [9] can simulate complex geographical structures, while `FAVITES` [10] can model social contact networks. Accounting for geographical and social structure complexity increases the realism of the simulations, but this increased realism comes at a substantial cost in terms of computational efficiency. For example, in our tests, simulating a pandemic up to 10^7 infected hosts with `nosoi` under a SIR-like model required

more than a day.

SANTA-SIM is a software that can simulate complex scenarios of pathogen genome sequence evolution including selection, recombination, and indels [11]. While SANTA-SIM therefore employ a very general models of genome evolution, the choice of epidemiological models is not as broad as typical simulators that are specifically developed to simulate epidemiological dynamics.

PhyDyn [12] and TiPS [14] are also packages with simulation capabilities and similar forward-backward idea to genealogies. In [14] it was shown that TiPS is at least an order of magnitude faster than PhyDyn, so we did a more detailed comparison with the former.

Next we provide a more detailed comparison of VGsim with MASTER [5] and TiPS [14] simulators.

9.1 MASTER

MASTER [5] is a general popular simulator which implements Gillespie algorithm, and it is built as a BEAST2 [15] package. It is used in many epidemiological studies [16–18]. In particular, in [19] MASTER was used for ABC inference, which suggests the potential use of VGsim for parameter estimation from real data.

MASTER does not have an option to sample individuals, instead it builds the full transmission chain of all the descendants of certain cases. This is a subset of VGsim's functionality, since the same behaviour can be achieved in our simulator by setting all recovery rates ρ_h to zero and all the sampling rates $c^k\zeta_h$ to the intended recovery rates. With these settings, all recovered individuals are sampled and each isolate appears in the resulting tree. We ran the same SIR model in both simulators with transmission rate $\lambda = 2.7$, and recovery/sampling rate $\zeta = 1$. One can see that VGsim is considerably faster and scales better with the simulation size 4. For 10 million samples MASTER failed with an out

of memory error on a machine with 16GB of memory. Moreover, by taking advantage of the fact that typically only a fraction of cases is sampled, and by efficiently sampling and storing only the genealogy of these samples instead of the whole epidemic transmission tree, `VGsim` allows to simulate large genealogies from massive pandemic scenarios.

`VGsim` also allows simulation scenarios not implemented in `MASTER`, such as the simulation of different haplotypes fitnesses and host immunity responses. Another new useful feature of `VGsim` is its intuitive and flexible interface. For example with 100 populations, 16 haplotypes and 3 immunity groups, there are approximately $5 \cdot 10^5$ reaction equations in the corresponding compartmental model.

`MASTER` has an option to approximate the simulated epidemiological dynamics with tau-leaping algorithm [20]. So, if the sample genealogy is not needed, `MASTER` should be the preferred way to simulate the epidemiological dynamics at least for simpler scenarios. For complicated models (especially with many populations), it is less clear if `VGsim` would be less effective compared to `MASTER` in simulating chains of events (only the forward simulation run) due to `VGsim`'s algorithm optimisations and caching of reaction rates. We did not yet explore this aspect in depth.

9.2 `TiPS`

The forward-backward approach is also used in the `TiPS` package [14], which generates sampled trees based on epidemiological trajectories. It allows a flexible approach for generating sampled subtrees by giving the user substantial choice in terms of times of sampling. For example, sampling times might depend on models parameters — i.e. be proportional to the change in number of recovered individuals, similar to the approach in `VGsim`. For the simplest SIR model `VGsim`

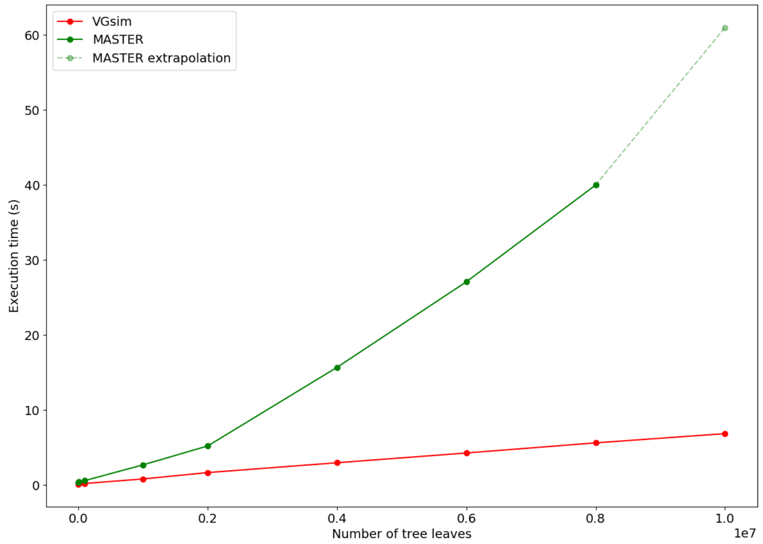

Figure 4: The comparison of `VGsim` and `MASTER` performance: the time to simulate a tree with a given number of leaves. `MASTER` crashed with an out of memory error for a tree with 10 million leaves, so we approximately extrapolated the final data point. The comparison was performed on a computer with Intel Core i7-7700K 4.20 GHz processor and 16GB of memory.

is about two times faster than `TiPS` with the direct Gillespie method (see Table 1). For the backward run, VGsim scales much better than `TiPS` (Table 2).

The focus of `VGsim` on complex epidemiological models is also captured in the software user interface, where `VGsim` allows a simplified specification of complex epidemiological dynamics.

Another important difference between the two simulators is the memory demand. We could not run `TiPS` for $3 \cdot 10^8$ iterations on our machines due to memory limitations, while on the same computers we could run `VGsim` for $2 \cdot 10^9$ iterations.

The strong point of `TiPS` is the implementation of hybrid and τ-leaping algorithms which can reduce the computation time and memory demand by

orders of magnitude. This is a feature that we would also like to include in `VGsim` in the future.

Number of iterations	VGsim	TiPS
10^6	0.19	0.31
$5 \cdot 10^6$	0.96	1.72
10^7	1.84	3.44
$5 \cdot 10^7$	9.87	17.57
10^8	19.06	38.94

Table 1: Forward run time in seconds for different number of iterations under the SIR model. The recovery rate is set to 1 and the transmission rate to 2.5. The tests were run on a MacBookPro with Quad-Core Intel Core i5 2 GHz processor and 16GB of memory.

Number of samples	VGsim	TiPS
10^4	0.059	242.4
$2 \cdot 10^4$	0.11	808.2
$3 \cdot 10^4$	0.18	1377.6
$4 \cdot 10^4$	0.22	1921.2
$5 \cdot 10^4$	0.27	3157.2

Table 2: Backward run time in seconds to generate genealogies for different sample sizes under the SIR model. The population size is 10^7 in all runs. The recovery rate is 1 while the transmission rate is 2.5. The tests were run on a MacBookPro with Quad-Core Intel Core i5 2 GHz processor and 16GB of memory.

The primary purpose of `VGsim` is to generate sample genealogies from complex epidemiological models, and was developed with particular attention to its computational efficiency so to scale efficiently of global-scale pandemic datasets. In addition, it can be used in other ways, for example, to generate random epidemiological trajectories, though there are ODE-based solutions such as `PhyDyn` that are better built for this specific purpose. To our knowledge, `VGsim` has a unique set of features when one needs to simulate an epidemiological model with a high level of complexity, including, but not limited to, the effects of selection, rare mutations, non-pharmaceutical interventions, and migrations.

References

[1] Gillespie DT. Stochastic Simulation of Chemical Kinetics. Annual Review of Physical Chemistry. 2007;58(1):35-55. PMID: 17037977. Available from: https://doi.org/10.1146/annurev.physchem.58.032806.104637.

[2] Thanh VH, Priami C, Zunino R. Efficient rejection-based simulation of biochemical reactions with stochastic noise and delays. The Journal of Chemical Physics. 2014;141(13):134116. Available from: https://doi.org/10.1063/1.4896985.

[3] Cao Y, Li H, Petzold L. Efficient formulation of the stochastic simulation algorithm for chemically reacting systems. The Journal of Chemical Physics. 2004;121(9):4059-67. Available from: https://doi.org/10.1063/1.1778376.

[4] McBroome J, Thornlow B, Hinrichs AS, De Maio N, Goldman N, Haussler D, et al. matUtils: Tools to Interpret and Manipulate Mutation Annotated Trees. bioRxiv. 2021. Available from: https://www.biorxiv.org/content/early/2021/04/04/2021.04.03.438321.

[5] Vaughan TG, Drummond AJ. A Stochastic Simulator of Birth–Death Master Equations with Application to Phylodynamics. Molecular Biology and Evolution. 2013 03;30(6):1480-93. Available from: https://doi.org/10.1093/molbev/mst057.

[6] Campbell F, Didelot X, Fitzjohn R, Ferguson N, Cori A, Jombart T. outbreaker2: a modular platform for outbreak reconstruction. BMC Bioinformatics. 2018;19(11):363. Available from: https://doi.org/10.1186/s12859-018-2330-z.

[7] Groendyke C, Welch D. epinet : An R Package to Analyze Epidemics Spread across Contact Networks. Journal of Statistical Software. 2018 02;83.

[8] Worby CJ, Read TD. 'SEEDY' (Simulation of Evolutionary and Epidemiological Dynamics): An R Package to Follow Accumulation of Within-Host Mutation in Pathogens. PLOS ONE. 2015 06;10(6):1-14. Available from: https://doi.org/10.1371/journal.pone.0129745.

[9] Lequime S, Bastide P, Dellicour S, Lemey P, Baele G. nosoi: A stochastic agent-based transmission chain simulation framework in R. Methods in Ecology and Evolution. 2020;11(8):1002-7. Available from: https://besjournals.onlinelibrary.wiley.com/doi/full/10.1111/2041-210X.13422.

[10] Moshiri N, Ragonnet-Cronin M, Wertheim JO, Mirarab S. FAVITES: simultaneous simulation of transmission networks, phylogenetic trees and sequences. Bioinformatics. 2018 11;35(11):1852-61. Available from: https://doi.org/10.1093/bioinformatics/bty921.

[11] Jariani A, Warth C, Deforche K, Libin P, Drummond AJ, Rambaut A, et al. SANTA-SIM: simulating viral sequence evolution dynamics under selection and recombination. Virus Evolution. 2019 03;5(1). Vez003. Available from: https://doi.org/10.1093/ve/vez003.

[12] Volz EM, Siveroni I. Bayesian phylodynamic inference with complex models. PLOS Computational Biology. 2018 11;14(11). Available from: https://doi.org/10.1371/journal.pcbi.1006546.

[13] Stadler T. Simulating Trees with a Fixed Number of Extant Species. Systematic Biology. 2011 04;60(5):676-84. Available from: https://doi.org/10.1093/sysbio/syr029.

[14] Danesh G, Saulnier E, Gascuel O, Choisy M, Alizon S. Simulating trajectories and phylogenies from population dynamics models with TiPS. bioRxiv. 2020. Available from: https://www.biorxiv.org/content/early/2020/11/09/2020.11.09.373795.

[15] Bouckaert R, Vaughan TG, Barido-Sottani J, Duchêne S, Fourment M, Gavryushkina A, et al. BEAST 2.5: An advanced software platform for Bayesian evolutionary analysis. PLOS Computational Biology. 2019 04;15(4):1-28. Available from: https://doi.org/10.1371/journal.pcbi.1006650.

[16] Kühnert D, Stadler T, Vaughan TG, Drummond AJ. Phylodynamics with Migration: A Computational Framework to Quantify Population Structure from Genomic Data. Molecular Biology and Evolution. 2016 04;33(8):2102-16. Available from: https://doi.org/10.1093/molbev/msw064.

[17] Poon AFY. Impacts and shortcomings of genetic clustering methods for infectious disease outbreaks. Virus Evolution. 2016 12;2(2). Vew031. Available from: https://doi.org/10.1093/ve/vew031.

[18] Volz EM, Didelot X. Modeling the Growth and Decline of Pathogen Effective Population Size Provides Insight into Epidemic Dynamics and Drivers of Antimicrobial Resistance. Systematic Biology. 2018 02;67(4):719-28. Available from: https://doi.org/10.1093/sysbio/syy007.

[19] Poon AFY. Phylodynamic Inference with Kernel ABC and Its Application to HIV Epidemiology. Molecular Biology and Evolution. 2015 05;32(9):2483-95. Available from: https://doi.org/10.1093/molbev/msv123.

[20] Gillespie DT. Approximate accelerated stochastic simulation of chemically reacting systems. The Journal of Chemical Physics. 2001;115(4):1716-33. Available from: https://doi.org/10.1063/1.1378322.
